# Mesencephalic trigeminal nucleus neurons with collaterals to both eyelid and masseter muscles shown by fluorescent double-labeling, revealing a potential mechanism for Marcus Gunn Syndrome

**DOI:** 10.1371/journal.pone.0293372

**Published:** 2023-11-07

**Authors:** Xue Shi, Jingdong Zhang, Gaiping Shi, Junyi Zhu

**Affiliations:** 1 Department of Anesthesiology, Hubei Key Laboratory of Geriatric Anesthesia & Perioperative Brain Health, and Wuhan Clinical Research Center for Geriatric Anesthesia, Tongji Hospital, Tongji Medical College, Huazhong University of Science and Technology, Wuhan, China; 2 Department of Anesthesiology, University of Cincinnati College of Medicine, Cincinnati, Ohio, United States of America; 3 Department of Stomatology, Union Hospital, Tongji Medical College, Huazhong University of Science and Technology, Wuhan, China; 4 School of Stomatology, Tongji Medical College, Huazhong University of Science and Technology, Wuhan, China; 5 Hubei Province Key Laboratory of Oral and Maxillofacial Development and Regeneration, Wuhan, China; Feinstein Institute for Medical Research Fertility Research Laboratory: Northwell Health Feinstein Institutes for Medical Research, UNITED STATES

## Abstract

Poking palpebral conjunctiva evoked upper-eyelid retraction during ophthalmic surgery. Iatrogenic eyelid ptosis occurred if eyelid branch of lachrymal nerve was sectioned. Mesencephalic trigeminal nucleus (Vme) neurons were labeled when tracer injected into lachrymal nerve innervating eyelid Mueller’s muscle. Masseter afferent Vme neurons projecting to oculomotor nucleus (III) was observed in toad and rat, which helps amphibians to stare prey when they open mouth widely to prey. We hypothesized single Vme neurons may have peripheral collaterals to both eyelid and masseter muscles. WGA-594 was injected into upper eyelid, and WGA-488 was simultaneously delivered into ipsilateral masseter muscle in the same rat. Then, double labeled Vme neurons were found under both conventional and confocal microscope. Meanwhile, contact of WGA-594 positive eyelid afferent Vme neurons with WGA-488 labeled masseter afferent ones were observed sometimes. Combined with our previous observation of oculomotor projection Vme neurons, we thought WGA-594/488 double labeled Vme cells, at least some of them, are oculomotor projecting ones. Contact between eyelid and masseter afferent Vme neurons are supposed to be electrotonically coupled, based on a line of previous studies. If exogenous or genetic factors make these Vme neurons misinterpret masseter input as eyelid afferent signals, these Vme neurons might feedforward massages to eyelid retractor motoneurons in the III. Besides, oculomotor projecting Vme neurons might be co-fired by adjacent masseter afferent Vme neurons through electrotonic coupling once the masseter muscle is activated. In these cases, Marcus Gunn Syndrome might occur. This finding leads to a new hypothesis for the Syndrome.

## Introduction

Marcus Gunn Syndrome (MGS) is usually observed in congenital ptosis and characterized by ptotic eyelid retraction concomitant with mouth opening and lateral shifting [1–4]. The MGS causes over curiosity and cosmetic disfigurement during social gathering [2, 5], and eventually develops amblyopia or anisometropia [3, 6, 7]. Its etiologic mechanism is still unclear; thought, there were a few hypotheses on the etiology [2–4, 8]. “Release hypothesis” is one of the hypotheses that has been long overlooked [8]. The key idea of release hypothesis is that MGS is a physiological reflex in lower animals but has been either concealed or extinguished during phylogenic development [3, 4, 8]. In human, the phylogenic old reflex is “released” as MGS in certain pathological circumstance [3, 4, 8]. The proposers of “release hypothesis” thought that basic neuronal circuit of that old reflex is preserved, to some extent, in higher animals. However, this hypothesis was based mainly on a neuronal circuit that remains disputed. The debated key question is whether or not there exits eyelid muscle primary afferent mesencephalic trigeminal nucleus (Vme) neuron?

It is well elucidated that Vme neurons innervate jaw muscle spindles and periodontium mechanoreceptors peripherally and their central processes widely project onto the brainstem and cervical spinal cord [9, 10]. A number of authors have shown primary extraocular muscle (EOM) afferent Vme neurons in monkey and cat, following injection of tracers into superior rectus (SR), levator palpebrae (LP) and medial rectus [11–13]. The number of EOM afferent Vme neurons are substantially fewer comparing to that of masticatory or periodontal afferent ones [11–13]. Meanwhile, typical structure of muscle spindle is not found in the EOM [14, 15]. Some authors failed to see any labeled primary EOM afferent Vme neuron in lamb, pig, rat *etc*. [16, 17]. These controversial results lead investigators who doubt the existence of EOM afferent Vme neuron to conclude that few labeled EOM afferent Vme neuron is resulted from spreading of tracers to surrounding orofacial muscles including temporal muscle.

While, in recent decades, a line of studies on rats, monkeys and even humans rekindled the argument on aforementioned topic [18–20]. Clinically, a group of ophthalmologists found that down-stretching eyelid palpebral conjunctiva evoked contraction of the LP during ophthalmic surgery, and iatrogenic ptosis occurred if eyelid branch of lachrymal nerve was removed or injured during the operation [19, 21–23]. This lachrymal nerve branch contains myelinated fibers exiting from trigeminal nerve, and electric stimulating this branch explicitly evoked a short latency response in the LP [19]. Hence, they thought the Mueller’s muscles act as a LP spindle and trigeminal proprioceptive fibers in this branch conduct the signals to the LP motoneurons [19, 21–23]. However, the key question is where these primary trigeminal proprioceptive afferent neurons are located—in the Vme [11–13] or in the trigeminal ganglion [16, 17]?

On the other hand, both implantation of tracers in the lachrymal gland and injection of trace into the Mueller’s muscle branch of lachrymal nerve gave rise to labeled somata in the Vme in the monkey and rat [18, 20]. The finding in monkey is consistent to the previous report [11]; while, the observation on rat by Fujita et al. [18] is a new finding. They further demonstrated that section of Mueller’s muscle branch of the lachrymal nerve caused ipsilateral ptosis on that rat. In light of aforementioned studies, we hypothesize that some Vme neurons may receive proprioceptive afferent signals simultaneously from both eyelid and masticatory muscle, similar to that some Vme neurons are simultaneously both masseter and periodontal afferent neurons [24]. To this end, we applied fluorescent tracers of two colors onto both eyelid and masseter muscles of the same rat, to see whether there would be any single Vme neuron, through its peripheral process’s collaterals, innervating both the eyelid and masseter muscles in the meantime. The potential linking of this anticipated finding with the MGS was discussed.

## Materials and methods

### Animals and drugs

Total 24 (half male/ half female) adult Sprague-Dawley rats (200–300 g) were used in the experiments. Surgical procedures and animal care were carried out in accordance with Chinese National Research Council’s Guide for the Care and Use of Laboratory Animals (in line with NIH Guide for Care and Use of animals). All efforts were made to minimize animal suffering and the number of animals used in the study.

Wheat germ agglutinin conjugated Alexa Fluor 594 and 488 (WGA-594 /488; Thermo Fisher Scientific, China LLC, Shanghai, Pudong) were prepared to 1.0 mg / ml in phosphate-buffer saline (1× PBS, Fisher Chemicals; pH 7.2–7.4) as stock solution. The stock solution was stored at -20°C before tracer injection, and final working solution was 50 μg/ml, diluted before injection.

### Single tract tracing of eyelid afferent Vme neurons

Five rats were intra-peritoneally (*i*.*p*.) administered atropine (0.15 mg/kg) and anesthetized with sodium pentobarbital (40 mg/kg, *i*.*p*.). WGA-594 of 5~7 μl was injected into eyelid using insulin syringe under deep anesthesia when no limb-withdrawal reflex was elicited by pinching the hind paw (S1A Fig). After either 12 h. or 24 h. survival, the animals were euthanized with overdose sodium pentobarbital (80–100 mg/kg, *i*.*p*.), and transcardially perfused with cold saline followed by 10% Formalin in 1× PBS (Fisher Chemicals, pH 7.2~7.4). The brainstems and tracer injected eyelid were removed, post-fixed overnight, and cryo-protected by 30% sucrose in 1× PBS. Coronal frozen sections of brainstem and sagittal section of eyelid, were cut in 14 μm-thickness and mounted on Plus^+^ pre-coated slides. Slides were sealed with Vectashield mounting medium (Vector Labs) for observations. Cell-count was carried out on every section that contains WGA-594 positive Vme neurons.

### Single tract tracing of masseter afferent Vme neurons

Similarly, three rats were used to perform the injection under the same anesthesia as described above. A skin incision was cut to expose the masseter muscle as previously described [25, 26]. The WGA-488 was injected, with a Hamilton micro-syringe, into the dorsal and ventral bellies of the masseter (about 15 μl per belly; S1B Fig). After either 12 h. or 24 h. survival, the animals were euthanized, perfused and processed in the same way as described above. Cell-count was performed on every other section.

### Double tract tracing of Vme neurons by simultaneous applying tracers to eyelid and masseter

Total 16 rats were given double injection of tracers into the eyelid (WGA-594) and masseter (WGA-488) simultaneously in the same way as aforementioned. WGA-594 and 488 double labeled Vme cells were observed and photographed under Nikon E-600 conventional fluorescent microscope. The better sections were selected and photographed under Nikon A1R Confocal Microscope using Fluorescein and Rhodamine set-up. The confocal imaging was processed through NIS-Elements AR software and converted in to TIFF file for manuscript preparation. All final figures were processed with Adobe Photoshop CS5.

### Statistical analyzing of labeled cells corresponding to injection sites

One-way ANOVA with Tukey and Newman-Kaul’s posttest for comparison of each column was used to compare all different number of 594 single and double labeled Vme cells corresponding to different type of injection, and to rate relative ratio of double labeled *vs* either 594 or 488 single positive cells as well. Statistical analysis was processed with Graph Pad Prism 5 (Graph Pad Software Inc. La Jolla, CA) and the difference were indicated as *p* < 0.05, marked by “*” in figures.

## Results

### Evidences of correct injections in the eyelid

#### Injection sites in the eyelid

**Fig 1:**
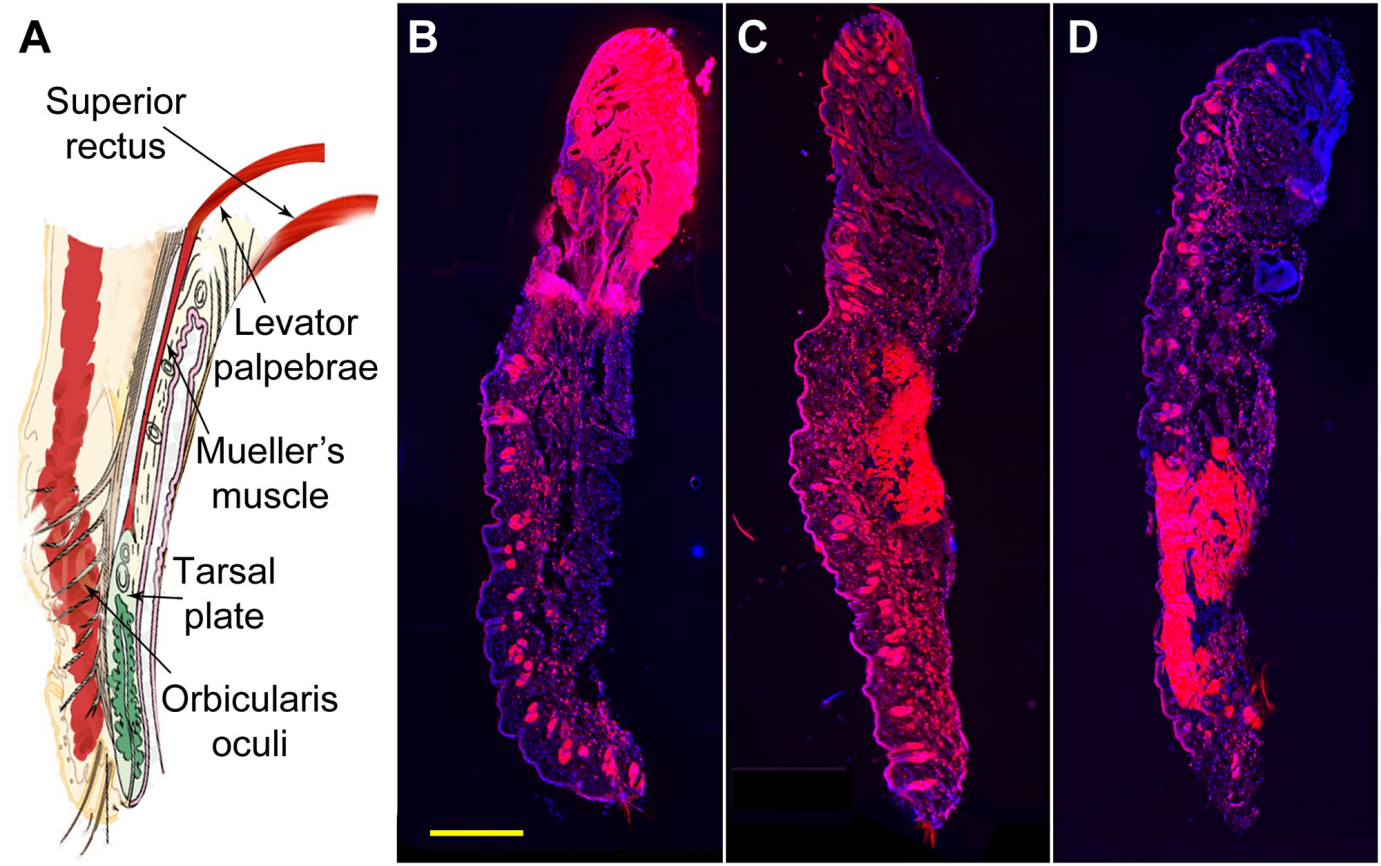
Classification of WGA-594 injection sites in the upper eyelid. **A**, the eyelid anatomy is schematically shown in order to match the injection site with the involved structures. **B**, type 1 injection site in dorsal part of the eyelid, the tracer appears to deeply spread to the SR and LP territory. **C**, type 2 injection site in middle portion of the eyelid, the dye stained area is apparently involving the Mueller’s muscle. **D**, type 3 injection site in ventral part of the eyelid, the distal part of the Mueller’s muscle seems to be stained by WGA-594. Scale bar = 500 μm.

WGA-594 injection sites in the eyelid could be divided in to 4 types. In type 1 site, the injection was deep enough to reach the SR and LP (Fig 1A and 1B), which resulted in a relatively higher number of labeled Vme neurons. In the type 2 site (Fig 1A and 1C), the tracer was located in middle part of the eyelid having Mueller’s muscle; while the type 3 site (Fig 1A and 1D) was close to distal part of the Mueller’s muscle. The number of labeled Vme cells in rats with types 2 and 3 injection sites was varied case by case, occasionally with zero labeling in the Vme. While, the type 4 site was either too superficial in the skin or only located in the edge of eyelid, generally giving rise to none of labeled cell in the Vme territory.

#### Retrograde labeled motoneurons in oculomotor nucleus (III)

**Fig 2:**
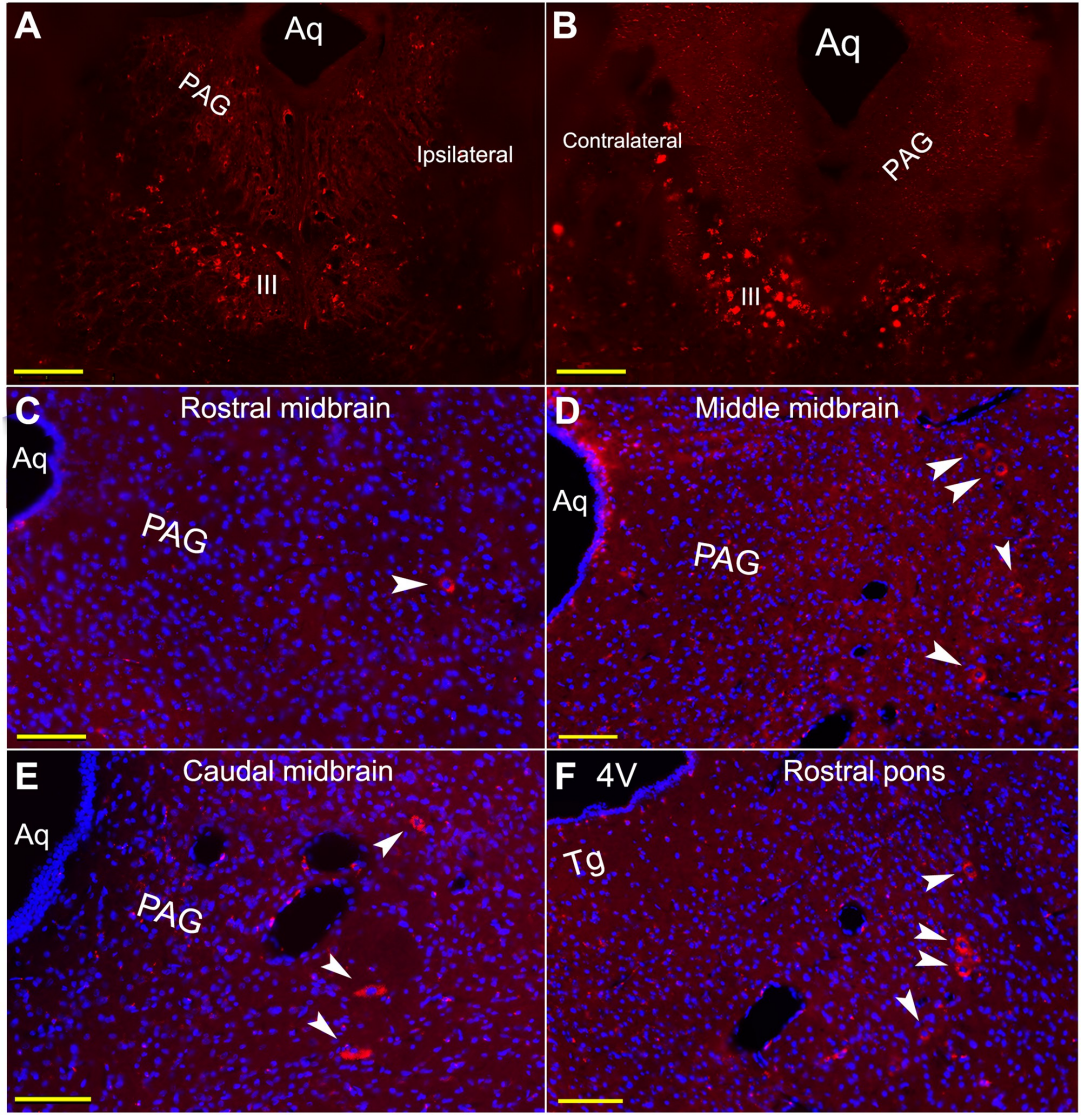
Retrogradely labeled III motoneurons and Vme neurons following injection of WGA-594 into the eyelid. **A**, labeled III motoneurons on coronal plane resulted from a type 1 injection. Most of them are situated in the III contralateral to tracer injection, suggesting they are SR motoneurons. **B**, similarly, on coronal plane of the middle III in the other rat, more labeled cells are located in contralateral nucleus with fewer labeled cells in ipsilateral side. Probably, some LP motoneurons among them are bilateral scattered. **C** ~ **F**, a number of Vme neurons are ipsilaterally distributed in rostral (**C**), middle (**B**) and caudal (**E**) midbrain, and at rostral pons (**F**). Aq, aqueduct; 4V, fourth ventricle; PAG, periaqueductal gray; scp, superior cerebellar peduncle; Tg, tegmentum. Scale bars = 200 μm at **A** and **B**; scale bars = 100 μm in **C** ~ **F**.

The other evidence was retrogradely labeled motoneurons in the III (Fig 2A and 2B). The labeled motoneurons were predominantly located in the middle column of the III contralateral to the injection (Fig 2A and 2B). This distributive pattern of labeling suggested the injection site involving the SR and LP, since this area of the III contains SR and LP motoneurons based on a couple of previous studies on distribution of EOM motoneurons in rats [27, 28].

### Single tract tracing of eyelid or masseter afferent Vme neurons

#### Distribution of labeled Vme neurons following injection of WGA-594 to eyelid

**Fig 3:**
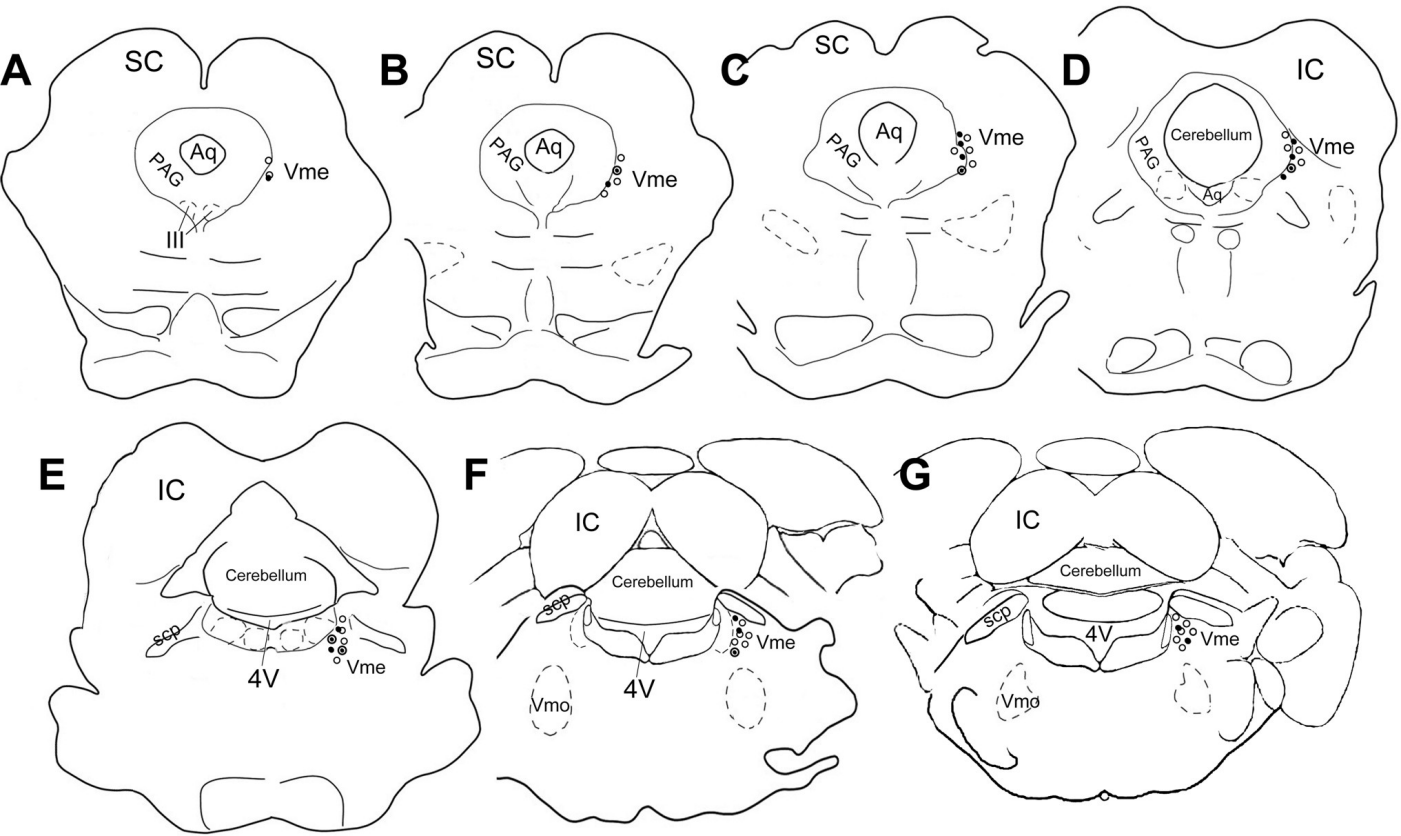
Schematic drawing of distribution of single and double stained Vme neurons. **A**, coronal sections at segments of rostral midbrain, filled circle represents 594 positive cell and opened circles are 488 labeled Vme neurons. Eccentric overlap of filled and opened circles indicates 594 and 488 labeled Vme cells coupled with each other. Each filled circle represents 1.5 ~ 3 eyelid-afferent Vme neurons; while, each opened circle represents 5 ~ 8 masseter-afferent Vme cells. **B** and **C**, coronal planes at middle portion of midbrain. Concentric overlap of filled and opened circle denotes 594 and 488 double labeled Vme neuron. **D** and **E**, showing caudal midbrain sections. **F** and **G**, displaying planes in rostral pons. IC, inferior colliculus; SC, superior colliculus; Vmo, trigeminal motor nucleus.

The WGA-594 labeled Vme neurons were observed scattering along the whole column of the Vme (Fig 2 arrowheads; Fig 3 filled circle), with more labeled cells in middle and caudal midbrain (Figs 2D, 2E and 3B–3D). The number of labeled Vme neurons seemed to depend more on the injection site (Fig 1) and less on survival time; nonetheless, more compactly labeled Vme neuronal somata were observed in 24 h survival cases. Thus, in subsequent studies, we allowed all animals to survive 24 h following dual tracer injection. Clear retrograde traced eyelid afferent Vme neurons were observed in about half (n = 11) of total (n = 21, five single injections and 16 dual injections) WGA-594 injection cases. Comparison among the injection sites in the eyelid, it appeared that the better results were commonly from the cases in which the tracer spread to the SR and LP, or to the deeper part of Mueller’s muscle (Figs 1A–1C and 2C–2F; 3 filled circles; Fig 5 arrowheads). Counting cells from all positive cases, we observed that 594 positive eyelids afferent Vme neurons were significantly more in rats with type 1 and 2 injection sites than that in animals with type 3 injection sites (Fig 6A, *p* < 0.05).

#### Distribution of labeled Vme neurons following masseter injection of WGA-488

**Fig 4:**
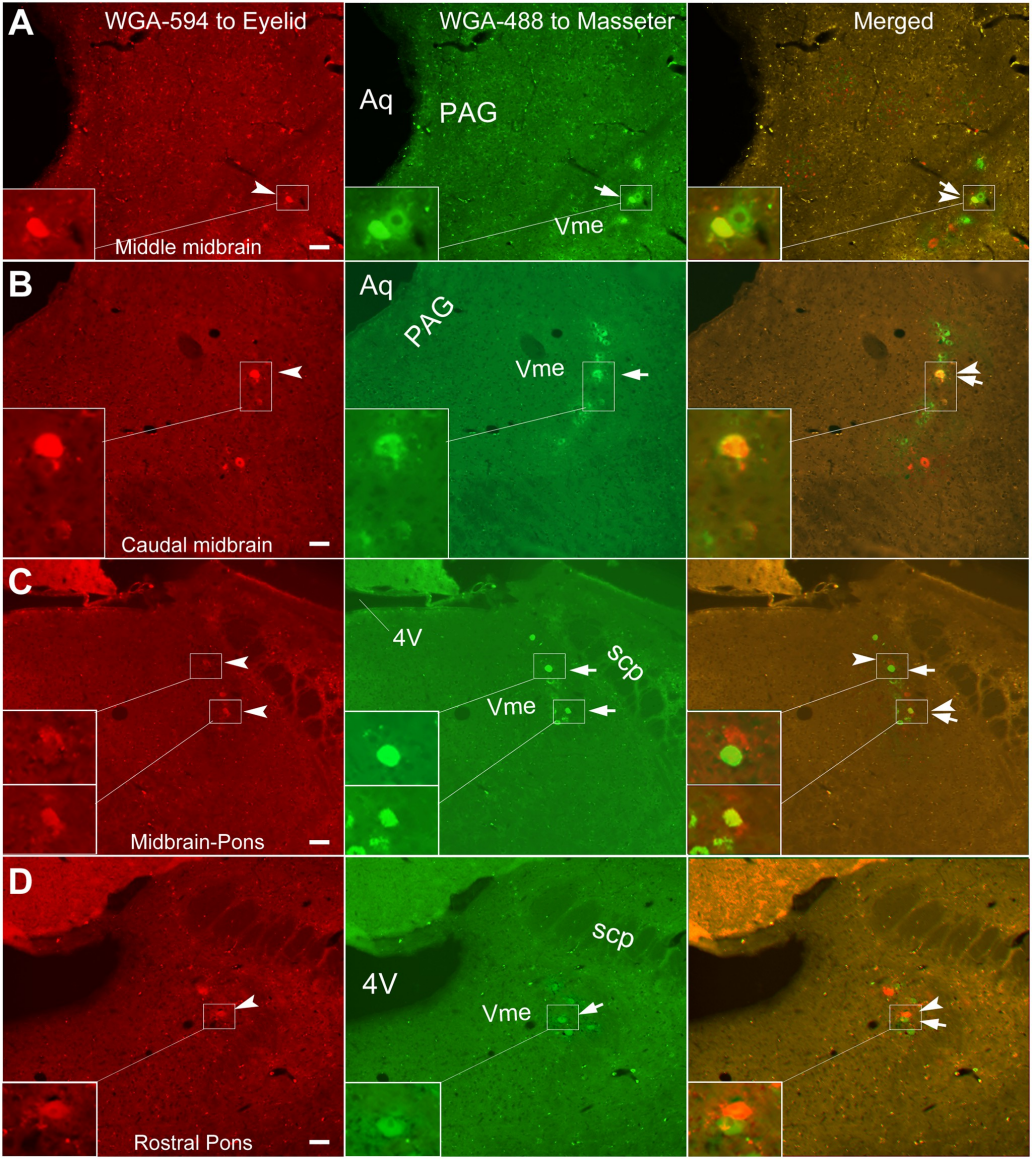
Representative images of WGA-594 and 488 double labeled Vme neurons. **A**, WGA-594 and 488 labeled Vme neurons are indicated by arrowhead and arrow, respectively; further, double labeled cell is marked with arrow-arrowhead. Insets exhibit magnified images, which clearly shows a 594–488 double labeled soma contact with a 488 single labeled one in the Vme at middle midbrain. **B**, showing a double labeled Vme neuron at caudal midbrain level. **C**, both contact between 594 and 488 positive soma and double labeled cell are visualized in the Vme (frame areas and insets) at conjunction of midbrain and pons. **D**, another contact between different dyes stained soma is envisioned in caudal Vme at pons (frame areas and insets). All scale bars = 50 μm.

**Fig 5:**
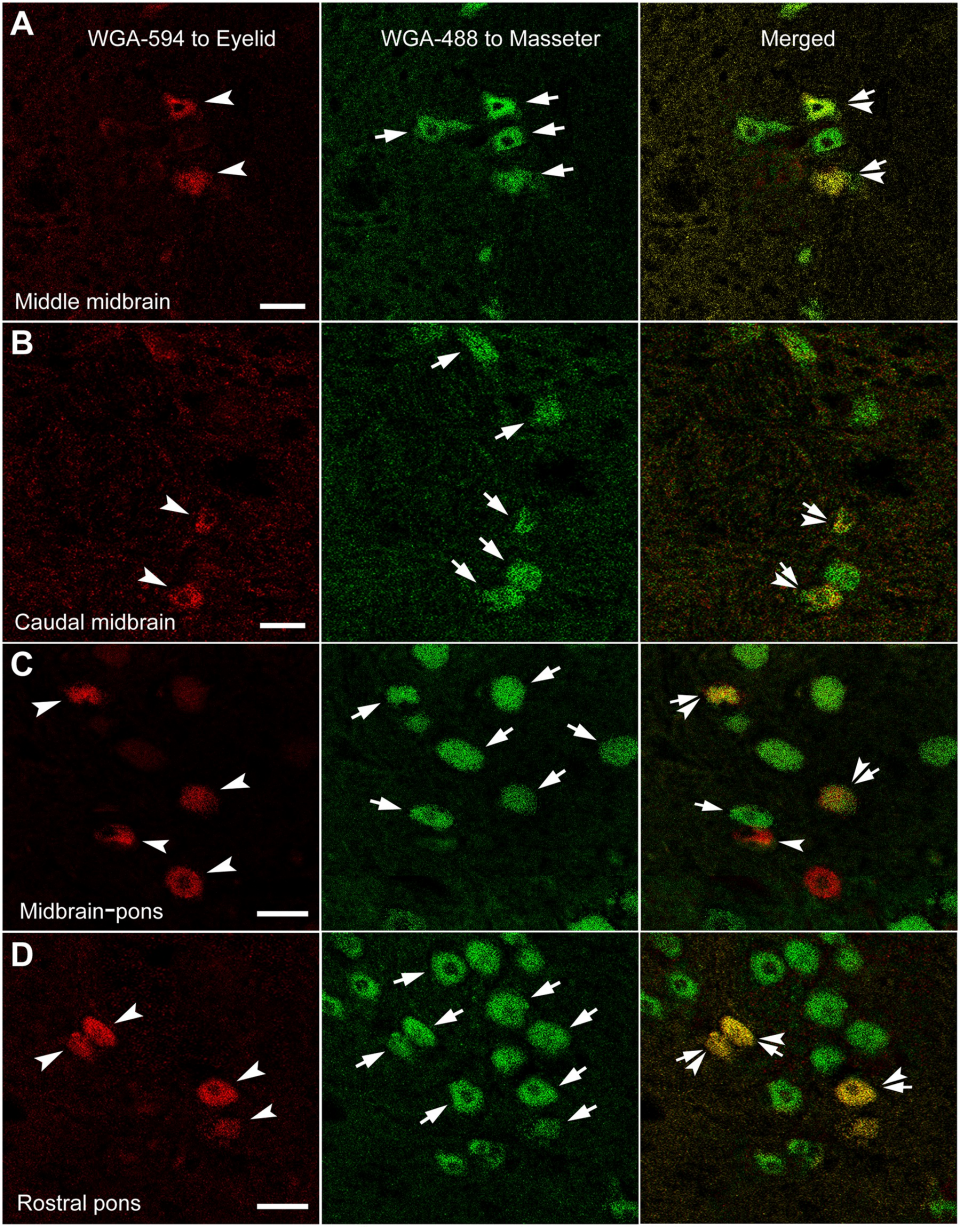
Confocal microscopic images of WGA-594 and 488 double labeled Vme neurons. In conformity, 594 positive eyelids afferent Vme neurons (arrowheads), 488 marked masseter afferent ones (arrows) and double labeled eyelid and masseter dual afferent Vme cells (arrows-arrowheads) are observed at middle (**A**), caudal midbrain (**B, C**) and rostral pons (**D**). Similarly, contacting of eyelid and masseter afferent Vme neurons with each other is seen from time to time (**B** and **C**). Also, a double labeled Vme soma contact to a 488 marked masseter afferent neuron is encountered (**B**). All scale bars = 50 μm.

The WGA-488 labeled Vme neurons were distributed along the whole column of the Vme with more WGA-488 traced cells situated in the pons, adjacent to the locus coeruleus (Fig 3 opened circles; Figs 4 and 5 arrows). The round or ovoid masseter afferent Vme neurons were visualized in all cases, either 12 h or 24 h after injection. The number of WGA-488 positive Vme neurons (Fig 3 opened circles) with DAPI marked nuclei were 382, 346 and 317, respectively.

### Double tract tracing of eyelid and masseter afferent Vme neurons

#### Pattern of WGA-594 and 488 double labeled Vme neurons

The co-localization of WGA-594 and 488 was identified in conventional microscope (Fig 4) and confirmed under laser scan confocal microscope (Fig 5). There seemed to be 3 kinds of relationship between WGA-594 and 488 co-localizations in the Vme: 1) exact co-localization of two fluorescent dyes in the same soma (Fig 3B–3F concentric overlapping of filled and opened circles; Fig 4A–4C arrow-arrowheads; Fig 5 arrow-arrowheads); 2) single (WGA-594 or 488) labeled somata contacting with the other single (WGA-488 or 594) labeled somata (Fig 3A and 3E–3G eccentric overlapping of filled and opened circles; Fig 4C and 4D Merged, frame area and inset; Fig 5C Merged); 3) double labeled somata contacting with 488 single labeled somata (Fig 4A frame and inset; Fig 5B).

#### Distribution of WGA-594 and 488 double labeled Vme neurons

The WGA-594 and 488 double labeled Vme neurons were distributed in entire midbrain and rostral pons (Figs 3–5), with more WGA-594 and 488 co-localized cells in the caudal midbrain (Fig 3A–3F). Some different dye single labeled Vme neurons were contacting each other at midbrain (Figs 3A; 4B and 4C) or rostral pons (Figs 3E–3G, 4C and 5C). Among them, double labeled Vme neurons contacting with adjacent 488 single labeled somata were occasionally visualized (Figs 4A and 5B).

**Fig 6:**
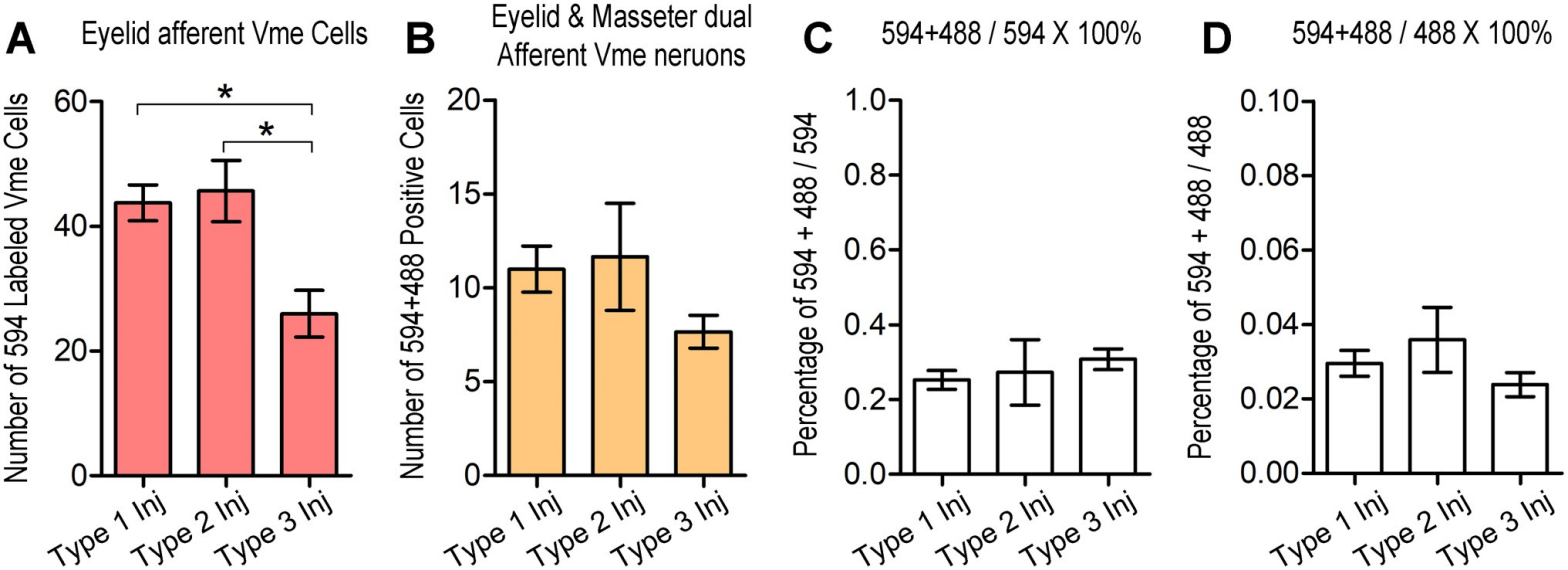
Comparison of number and ratio of labeled Vme cells corresponding to type of injection. **A**, 594 positive Vme neurons in rats with type 1 and 2 injection sites are significantly more than that with type 3 injection (*p* < 0.05). **B**, 594 **+** 488 co-labeled Vme cells corresponding to different type of injection show no significant difference. **C**, there is about 20–30% of 594 + 488 double labeled Vme neurons among 594 positive eyelids afferent ones, but no statistical difference is shown between cases with different type of injection site. **D**, around 2–4% of 594 + 488 co-labeled cells is observed among all 488 labeled masseter afferent Vme cells; similarly, there is no statistical difference between cases with different type of injection site.

Similarly, relationship between type of injection sites and number of double labeled Vme neurons was examined and compared. While, statistical comparison showed no significant difference in number of double labeled Vme cells corresponding to that 3 types of injection sites (Fig 6B). In addition, the ratio of 594 and 488 co-labeled Vme neurons *vs* either 594 or 488 single labeled Vme cells corresponding to types of injection sites were also statistically compared, but no significant difference was identified among the 3 injection site cases (Fig 6C and 6D). Apart from, about 20 ~ 30% of 594 positive Vme neurons were co-localized with 488 labeled Vme cells; whereas, around 2 ~ 4% of 488 positive Vme neurons were co-localized with 594 labeled ones (Fig 6C and 6D). Note, number of contacting, instead of co-localization, between 594 and 488 single labeled Vme cells were not included in aforementioned analysis, because it is hard to distinguish whether the 594 and 488 positive cells are contacting or stacking each other under light microscope. Nonetheless, these contacting between 594 and 488 labeling has been qualitatively verified under confocal microscopic examination (Fig 5).

## Discussion

### Technique consideration

As mentioned in introduction, there seems to be some species difference about the existence of EOM primary afferent Vme neuron. Previous observation of EOM afferent Vme neurons was reported in monkey and cat [11–13]; whereas, the negative finding was reported in lamb, pig, rat *etc*. [16, 17]. The reports on presence of EOM afferent Vme neurons in cats were consistent in all previous studies [12, 13, 16], but not in monkey [11, 29]. Therefore, the other reason than specie difference may exist. The tracer used by authors is probably one of the causes for variated results, reflected by comparison of these published protocols. In the studies on cats, all authors applied either horseradish peroxidase (HRP) or WGA conjugated HPR. But in monkey’s work, the presence of Vme neurons was resulted when investigators injected WGA-HRP into either SR or LP [11], or orbital part of the lacrimal gland [20]. However, in the other study on the monkey, the authors placed a piece of dental sponge soaked with diamidino yellow solution onto the LP and SR, which showed no labeling in the Vme [29]. Accordingly, it is rational to consider that variance of the results is due to the using of different tracers. Indeed, a methodological study on comparison of different retrograde tracers demonstrated that WGA is the most efficient one but the diamidino yellow is about the least effective tracer, and the HRP is in between [30].

Therefore, we chose WGA conjugated Alexa Fluor as retrograde tracer to re-examine the innervation of Vme neurons onto the SR/LP in current work. Originally, researchers conjugated WGA with radioactive iodine^125^, which displayed a conspicuous outcome of retrograde labeling compared to the HRP and early natural fluorescent dyes [30, 31]. Nowadays, WGA conjugated Alexa Fluor, much easier to process, has been extensively used in neuronal tract tracing studies in central and peripheral nervous system [30, 31]. WGA is a lectin-based molecule and isolated from the wheat, *Triticum vulgaris*. It specifically binds to a set of molecules that are ubiquitously anchored on neuronal membranes [30, 31]. Both WGA and HRP are internalized by neurons or axon boutons during uptake; while, higher affinity of WGA to cell membrane makes WGA to be a super neuronal tracer comparing to the other ones [30, 31]. This is also verified by comparison of the number of WGA-488 labeled masseter afferent Vme neurons in current work with the number of fluorescent dye and HRP labeled ones in our previous studies. In these early works [24, 32], the number of masseter afferent Vme neurons were 200 ~ 300, following injection of fluorescent dye and of HRP into the masseter nerve of adult rats. In the current work, by using the same strain and size of animals, the number of masseter afferent Vme neurons were 300 ~ 382 following injection of WGA-488 into the masseter muscle. Notably, nerve injection of tracer commonly generates higher number of labeled neurons than muscle delivery [33].

On the other hand, the number of 594 labeled eyelid afferent Vme neurons is relatively higher in our current work, from 16 to 54 with averagely 37, comparing to HRP labeled ones of about 12 in monkey and 25 or more in cat [11, 12]. Though, the previous authors did not count all sections; *e*.*g*., the authors those injected tracers to monkey’s eyelid counted about ¼ of their sections containing the Vme [11]. We have compared the number of 594 positive cells in rats with different type of injection site and noted that the number is significantly higher in cases with type 1 and 2 injection sites than that from type 3 injection. Whereas, 594 and 488 co-labeled Vme cells from different type of injection were not obviously different, neither the ratio of double labeled cells *vs* single labeled Vme neurons (see Fig 6). One possible reason is, we considered, that more nerve fibers from transvers branch of lachrymal nerve [19, 22] were exposed to the tracers once the needle tip stabbed that nerve branch during type 1 and 2 injections. While, type 3 injection is not deeper enough to break that nerve branch. The other possibility is that SR, LP and/or deeper Mueller’s muscle contain more palisade endings [14–16] that are innervated by eyelid branch of lachrymal nerve and connected to the Vme [11, 12].

### Significance of finding eyelid and masseter dual afferent Vme neuron

**Fig 7:**
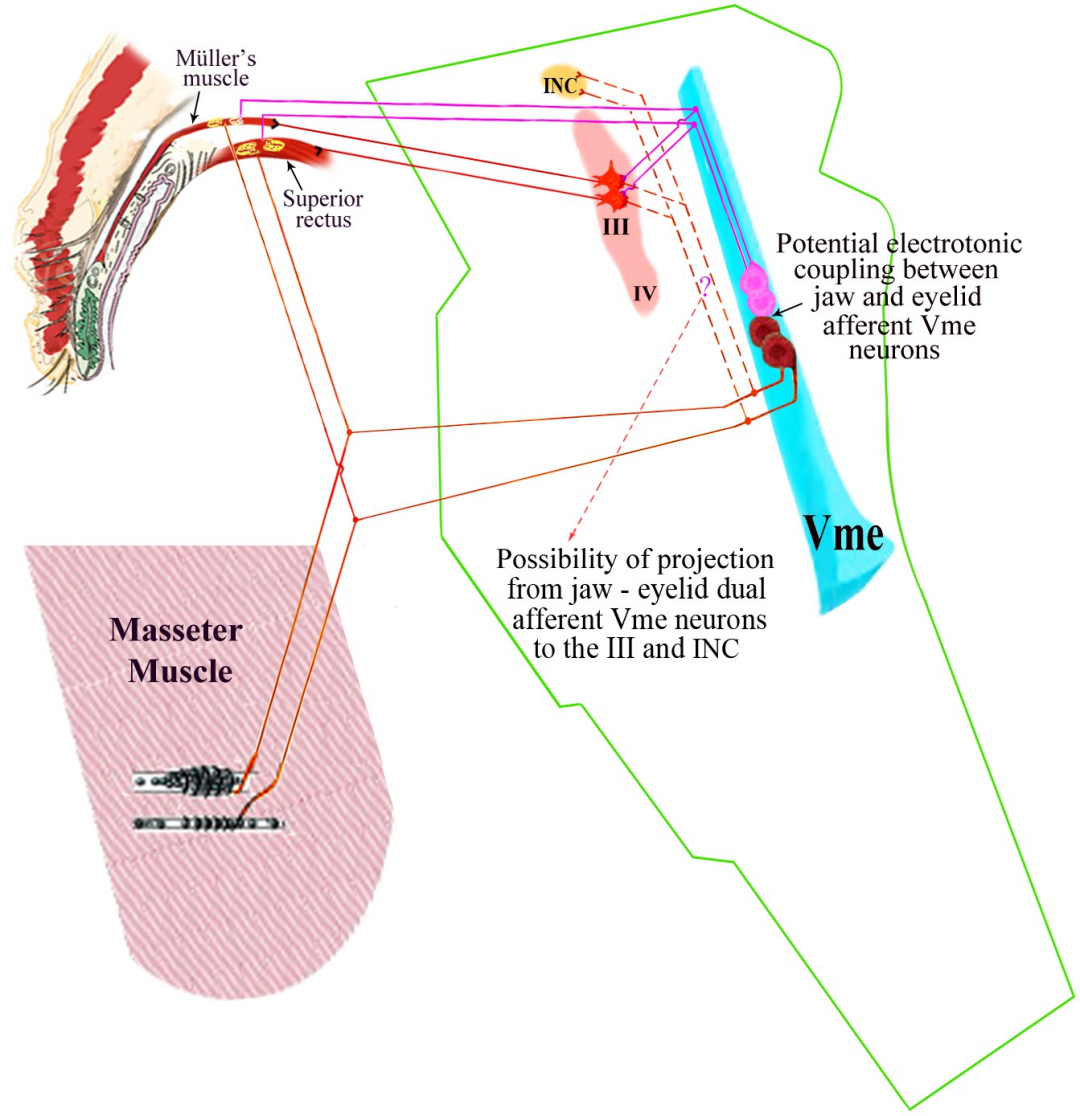
Schematic summary of the neuronal circuit underling the mechanism of MGS. Pink circuit represents single eyelid muscle afferent Vme neurons with their peripheral and central processes trajectory. Brown circuit with dotted line shows eyelid and masseter muscle dual afferent Vme neurons and the potential trajectories of their peripheral and central processes. INC is interstitial nucleus of Cajal, a nucleus contains premotor neurons to the III and IV, and a preoculomotor center controls vertical—torsional eye movements. For the masseter afferent Vme neurons those do not directly project to the III/IV and INC (as if removing dotted lines), there may exist an electrotonic coupling of them with neighbor eyelid afferent Vme neurons (arrow and elucidation). Somatofugal action potential may discharge from an eyelid afferent Vme neuron (pink) once its resting membrane potential reached to a threshold, through an electrotonic coupling to an adjacent masseter afferent Vme neuron (brown) if it is highly activated by jaw movements.

The present work added new evidence to support the existence of primary EOM afferent, and/or eyelid afferent Vme neurons innervating Mueller’s muscle [11–13, 18]. Based on a serial of studies in recent decades, Mueller’s muscle mechanoreceptors play a major role to sense gravity and elevate upper eyelid, without excluding the function of palisade receptors in the SR and LP [18–23]. Some electrophysiological recording and neural tract tracing studies exhibited that the related proprioceptive primary afferent neurons are located in the Vme [18–20]. Yet, they did not show through which pathway these Vme neurons relayed proprioceptive information to the III motoneurons. Fujita et al. [18] proposed frontalis muscle participating in the reflex to elevate eyebrow and eyelid, without excluding participation of the SR and LP. Whereas, Zhang and colleagues demonstrated that central axons of the Vme neurons directly project to III motoneurons and their premotor neurons by means of neuronal tract tracing and electrophysiological recording in rats [25, 26]. The authors thought these III projecting Vme neurons may contain the eyelid afferent Vme neurons; if so, the reflexive arc is closed (Fig 7).

However, when the authors injected tracers to the caudal Vme in aforementioned studies [25, 26], it is apparent that all Vme neurons in the injection site, possibly including eyelid, masticatory and periodontal afferent Vme neurons would be all traced, since there has been no functional compartmentation of the Vme neurons reported. Thus, is it possible for a single Vme neuron to receive afferent signals from both the eyelid and masticatory muscles? Our current work has clearly said “yes” to this question. In addition, we also observed that some somata of eyelid and masseter afferent Vme neuron contacted with each other (*e*.*g*. Figs 4A, 4C, 4D, 5B and 5C), and they might be electrotonically coupled through gap junctions [34, 35]. But, what would be the functional profile of these double origin afferent Vme neurons and their electrotonic coupling? This might be related to a neuronal circuit (Fig 7) underling the mechanism of MGS.

### Hypothesis for normal subjects and MGS cases

What role of these Vme neurons that receive both eyelid and masseter afferent signals may play in normal mammals is currently unknown, but it might be an add-on for a masticatory oculomotor reflex in amphibian [4, 8, 36]. Direct projections from masticatory primary afferent Vme neurons to the III was observed in *Xenopus toad* [36]. This neuronal circuit may assistant a *toad* to stare on its prey when it opens mouth widely to prey [4, 8, 36]. This kind of reaction was considered as “primitive reflex” [2, 4, 8, 25] and may be gradually extinguished during phylogenic development. If a single Vme neuron simultaneously receives both eyelid and masseter afferent inputs in amphibian, the excitability of this Vme neuron would be enhanced when the masticatory muscles are stretched or contracted. Therefore, the single Vme neuron receiving both eyelid and masseter afferent inputs is probably an add-on circuit for amphibian to synergize that “primitive masticatory oculomotor reflex”. But mammals or humans do not need this reflex to help them to prey; hence, we assumed that the neuronal circuit in rats [25, 26] is possibly a kind of structural residue for that extinguished “reflex”.

The facial behavior of MGS in human furthered the assumption that aforementioned neuronal circuit residue is preserved in human as well. A hallmark symptom of MGS is that ptotic eyelid could not be voluntarily elevated during gaze, but is able to be retracted involuntarily by mouth opening and/or lateral shifting [2–4]. Clinic studies on the MGS had revealed that stimulating pterygoid nerve branch can elicit ipsilateral eyelid retraction, and section of the nerve from the trigeminal motor root could relieve the upper eyelid activity [2, 37], supporting the existence of that neuronal circuit residue in human. Wartenberg, an early proposer of “release hypothesis”, stated in his literature: “An associated movement on forceful opening of the mouth, consisting of simultaneous wide opening of the eyes and spreading of the fingers, is found in children”. And he thought “children with photophobia involuntarily open their mouths in trying to open their eyes” as “an incidence of phylogenic old reflex that is not necessarily due to the photophobia” [4]. Obviously, he believed that “primitive reflex” like behavior would occasionally show-up, especially in children. Accordingly, the structural residue of old neuronal circuit conducting MGS behavior is actually retained in healthy human. This idea is supported by more evidences after Wartenbarg’s early publication: 1) Some cases of MGS occur at school age without presage, instead of in-born [2], and some pediatric MGS cases are self-healing during growing-up, or with a pattern of alternative healing and relapsing [2, 4]. 2) Acquired MGS was occasionally occurred following brain trauma or tumor, and was either recovered or being long lasted until loss of tracing [2, 4, 8]. 3) Retraction of the eyelid was evoked by stimulation of trigeminal motor root during microvascular decompressing surgery in cases of trigeminal neuralgia, in those otherwise are completely healthy subjects [38]. 4) Electromyography responses in upper eyelid was recorded in healthy human volunteers when they perform static forceful occlusion with the mouth closing and the face still, reflecting isometric contraction of masseter muscle could somehow activate eyelid elevator muscle [39].

Therefore, we considered that primitive masticatory oculomotor reflex in high taxonomic rank animal might be suppressed or concealed by more frequently and actively used performance that is conducted by advanced and newly-developed networks [2, 4, 8]. The looked abnormal behavior would be released or re-manifested if the advanced brain network becomes disordered. Normally, the Vme neurons receiving both eyelid and masseter afferent inputs would relay proprioceptive afferent signals to their correct motor servo, so the jaw-eyelid synkinesis would not occur or just occasionally show-up as delineated by Wartenbarg [4]. The correct reflexes may have been established through thousands of practices during individual development, and the primitive reaction of eyelid muscle to masticatory muscle afferent would be suppressed or inhibited effectively. Whenever the new-established correct reflexes, and/or inhibition on the old one was interrupted by exogenous forces, *e*.*g*. trauma or tumor, or *via* genetic misleading, the jaw-eyelid synkinesis would be manifested as observed in the MGS. On the other hand, the masseter afferent Vme neurons are probably electrotonically coupled with the eyelid afferent ones through gap junctions (Fig 7 between pink and brown somata). Fujita et al. [18] had uncovered that eyelid afferent Vme neurons communicated with each other, or with different primary afferent Vme neurons, by using gap junction permeable dye. Significantly, somatofugal action potentials have been recorded from Vme neurons those coupling to the other discharging ones, once the former’s resting membrane potential reached to a threshold able to initiate outbound action potentials [34, 35]. Thus, discharge of masseter afferent Vme neurons would probably co-fire the neighbor eyelid afferent Vme neurons, and consequently excite the LP/SR motoneuron through the III projecting Vme neurons to motivate a jaw-eyelid synkinesis—the hallmark facial behavior of the MGS.

## Supporting information

S1 Fig Injection of WGA-Alexa dye into upper eyelid and masseter muscles. **A**, an insulin syringe with 31-gauge needle was used to inject WGA-594 (5–7 μl per eyelid) into upper eyelid. The needle was inserted through inner surface of the eyelid slightly above the eyelid edge. **B**, the 25-μl Hamilton microsyringe with 22-gauge needle was used to introduce WGA-488 into dorsal and ventral belly (15–20 μl per belly) of the masseter muscle.
(TIF)

S1 Table Number of WGA 594 labeled Vme neurons in 4 type of injection. (DOCX)

S2 Table Number of 594+488 double labeled Vme cells in 4 type of injection. (DOCX)

S3 Table Double labeled Vme cells and percentage of double *vs* single labeled ones. (DOCX)
