# Supplementary figures and images for: Mesencephalic trigeminal nucleus neurons with collaterals to both eyelid and masseter muscles shown by fluorescent double-labeling, revealing a potential mechanism for Marcus Gunn Syndrome
Source: PLoS One. 2023 Nov 7;18(11):e0293372. doi: 10.1371/journal.pone.0293372 (PMC10629631; doi:10.1371/journal.pone.0293372)

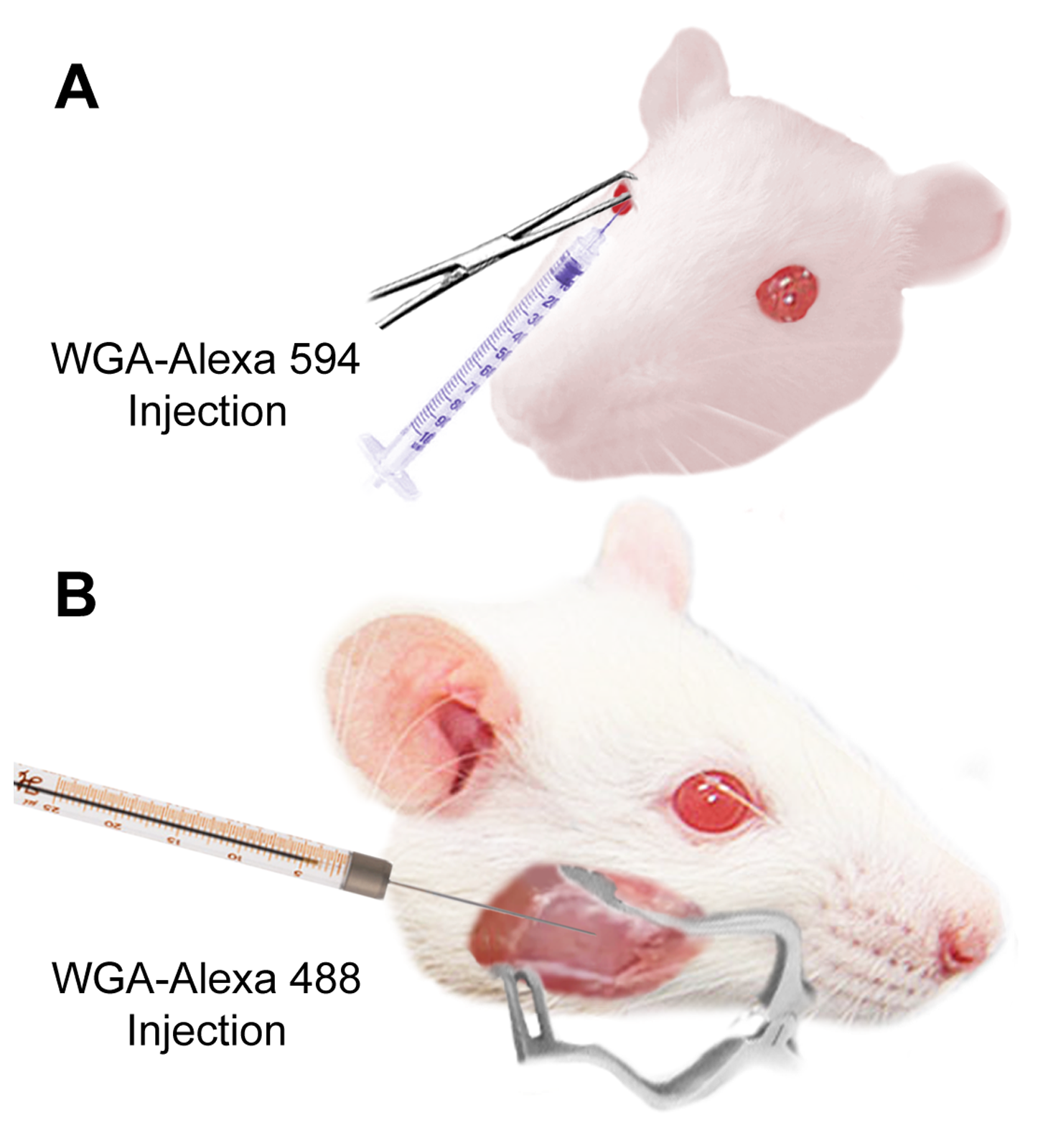

Supplement: S1 Fig — A, an insulin syringe with 31-gauge needle was used to inject WGA-594 (5–7 μl per eyelid) into upper eyelid. The needle was inserted through inner surface of the eyelid slightly above the eyelid edge. B, the 25-μl Hamilton microsyringe with 22-gauge needle was used to introduce WGA-488 into dorsal and ventral belly (15–20 μl per belly) of the masseter muscle. (TIF) [file pone.0293372.s001.tif]
