# Supplementary material for: Mesencephalic trigeminal nucleus neurons with collaterals to both eyelid and masseter muscles shown by fluorescent double-labeling, revealing a potential mechanism for Marcus Gunn Syndrome
Source: PLoS One. 2023 Nov 7;18(11):e0293372. doi: 10.1371/journal.pone.0293372 (PMC10629631; doi:10.1371/journal.pone.0293372)
Supplement: S1 Table — (DOCX) [file pone.0293372.s002.docx]

**S1 Table. Number of WGA 594 Labeled Vme Neurons in 4 Type of Injection**

| Type of Injection | Number of 594 Labeled Vme Neurons | | | | | | | Mean ± SD |
| --- | --- | --- | --- | --- | --- | --- | --- | --- |
| Type 1 (7 cases) | 55 | 49 | 51 | 42 | 31 | 48 | 35 | 44.43 ± 8.79 |
| Type 2 (4 cases) | 47 | 32 | 44 | 59 |  |  |  | 45.50 ± 11.09 |
| Type 3 (5 cases) | 35 | 18 | 16 | 38 | 21 |  |  | 25.60 ± 10.16 |
| Type 4 (5 cases) | 0 | 3 | 0 | 0 | 0 |  |  |  |
