# Supplementary material for: Mesencephalic trigeminal nucleus neurons with collaterals to both eyelid and masseter muscles shown by fluorescent double-labeling, revealing a potential mechanism for Marcus Gunn Syndrome
Source: PLoS One. 2023 Nov 7;18(11):e0293372. doi: 10.1371/journal.pone.0293372 (PMC10629631; doi:10.1371/journal.pone.0293372)
Supplement: S2 Table — (DOCX) [file pone.0293372.s003.docx]

**S2 Table. Number of 594+488 Double Labeled Vme Cells in 4 Type of Injection**

| Type of Injection | Number of 594+488 Labeled Vme Neurons | | | | | | Mean ± SD |
| --- | --- | --- | --- | --- | --- | --- | --- |
| Type 1 (5 cases) | 9 | 15 | 12 | 8 | 11 |  | 11.00 ± 2.74 |
| Type 2 (3 cases) | 14 | 6 | 15 |  |  |  | 11.67 ± 4.93 |
| Type 3 (6 cases) | 7 | 5 | 6 | 11 | 9 | 8 | 7.67 ± 2.16 |
| Type 4 (2 cases) | 0 | 0 |  |  |  |  |  |
