# Supplementary material for: Mesencephalic trigeminal nucleus neurons with collaterals to both eyelid and masseter muscles shown by fluorescent double-labeling, revealing a potential mechanism for Marcus Gunn Syndrome
Source: PLoS One. 2023 Nov 7;18(11):e0293372. doi: 10.1371/journal.pone.0293372 (PMC10629631; doi:10.1371/journal.pone.0293372)
Supplement: S3 Table — (DOCX) [file pone.0293372.s004.docx]

**S3 Table**. **Double Labeled Vme Cells and Percentage of Double *vs* Single Labeled Ones**

| Injection site type | 594+488 | Single 594 | 594+488/594  (ratio) | 594+488/594  (percentage) | 594+488 | Single 488 | 594+488/488  (ratio) | 594+488/488  (percentage) |
| --- | --- | --- | --- | --- | --- | --- | --- | --- |
| Type 1 | 11 ± 3 | 44 ± 9 | 11/44 | 25% | 11 ± 3 | 373 ± 15 | 11/373 | 3% |
| Type 2 | 12 ± 5 | 46 ± 11 | 12/46 | 27% | 12 ± 5 | 325 ± 19 | 12/325 | 4% |
| Type 3 | 8 ± 2 | 26 ± 10 | 8/26 | 31% | 8 ± 2 | 332 ± 19 | 8/332 | 2% |
